# Multidrug-resistance and extended-spectrum beta-lactamase-producing lactose-fermenting enterobacteriaceae in the human-dairy interface in northwest Ethiopia

**DOI:** 10.1371/journal.pone.0303872

**Published:** 2024-05-21

**Authors:** Achenef Melaku Beyene, Mucheye Gizachew, Ahmed E. Yousef, Hana Haileyesus, Ahmed G. Abdelhamid, Adugna Berju, Meseret Molu Tebeje, Tigest Feleke, Baye Gelaw

**Affiliations:** 1 Department of Medical Microbiology, School of Biomedical and Laboratory Sciences, College of Medicine and Health Sciences, University of Gondar, Gondar, Ethiopia; 2 Department of Food Science and Technology, Ohio State; University, Ohio, Columbus, United States of America; 3 College of Veterinary Medicine and Animal Sciences, University of Gondar, Gondar, Ethiopia; 4 Clinical Bacteriology Unit, Comprehensive Specialized Teaching Hospital, University of Gondar, Gondar, Ethiopia; Bangladesh Agricultural University, BANGLADESH

## Abstract

**Background:**

Antimicrobial resistance (AMR) is among the top public health concerns in the globe. Estimating the prevalence of multidrug resistance (MDR), MDR index (MDR-I) and extended-spectrum beta-lactamase (ESBL)-producing lactose fermenting Enterobacteriaceae (LFE) is important in designing strategies to combat AMR. Thus, this study was designed to determine the status of MDR, MDR-I and ESBL-producing LFE isolated from the human-dairy interface in the northwestern part of Ethiopia, where such information is lacking.

**Methodology:**

A cross-sectional study was conducted from June 2022 to August 2023 by analyzing 362 samples consisting of raw pooled milk (58), milk container swabs (58), milker’s hand swabs (58), farm sewage (57), milker’s stool (47), and cow’s feces (84). The samples were analyzed using standard bacteriological methods. The antimicrobial susceptibility patterns and ESBL production ability of the LFE isolates were screened using the Kirby-Bauer disk diffusion method, and candidate isolates passing the screening criteria were phenotypically confirmed by using cefotaxime (30 μg) and cefotaxime /clavulanic acid (30 μg/10 μg) combined-disk diffusion test. The isolates were further characterized genotypically using multiplex polymerase chain reaction targeting the three ESBL-encoding- genes namely *bla*_*TEM*_, *bla*_*SHV*_, and *bla*_*CTX-M*._

**Results:**

A total of 375 bacterial isolates were identified and the proportion of MDR and ESBL-producing bacterial isolates were 70.7 and 21.3%, respectively. The MDR-I varied from 0.0 to 0.81 with an average of 0.30. The ESBL production was detected in all sample types. Genotypically, the majority of the isolates (97.5%), which were positive on the phenotypic test, were carrying one or more of the three genes.

**Conclusion:**

A high proportion of the bacterial isolates were MDR; had high MDR-I and were positive for ESBL production. The findings provide evidence that the human-dairy interface is one of the important reservoirs of AMR traits. Therefore, the implementation of AMR mitigation strategies is highly needed in the area.

## Introduction

Enterobacteriaceae is a diverse family of Gram-negative, facultative anaerobic, rod-shaped and non-spore-forming bacteria and broadly divided as lactose fermenters and non-fermenters. The lactose-fermenting members of Enterobacteriaceae (LFE) are commonly termed coliforms, which are a group of bacteria that can induce mainly opportunistic infections [1–3]. However, LFE also includes primary pathogenic serotypes of *E*. *coli*, which cause more than one million illnesses in the world annually. Particularly, Shiga toxin-producing *E*. *coli* induces bloody diarrhea and could lead to the hemolytic uremic syndrome, a disease that often includes acute kidney failure [4]. Additionally, LFE can carry antimicrobial resistance (AMR) traits that are transmissible to other pathogenic bacteria [5].

Antimicrobial resistance is becoming an immense public health problem globally. It has been estimated that more than 700,000 people die annually worldwide due to infections caused by AMR pathogens. If no action is taken, the problem will grow and worsen in the future. The predicted estimate showed that AMR may induce the death of 10 million people globally towards 2050 [6]. The impact is very high in low-income countries (LIC) due to the high prevalence of infectious diseases, irrational uses and over-the-counter availability of antimicrobials, pollutants in the environment, agricultural residues, instability, displacement or migration of people and animals, and absence of sufficient laboratories for isolation and antimicrobial susceptibility testing, and regular surveillance [7]. Studies showed that people in LIC are more likely to die from AMR than those in high-income countries [7, 8]. The World Bank indicated that AMR will elevate the rate of poverty and it is one of the obstacles to achieve sustainable development goals. Studies also showed that AMR will result in annual losses of about 7% of the gross domestic products in LIC by 2050 [7–9].

A bacterium may be resistant to one antimicrobial agent or more. However, the tendency to resist more than two classes of antimicrobial agents (AMAs), which is termed multidrug resistance (MDR), is becoming a common phenomenon. The MDR character limits treatment options and aggravates the problem of AMR [10, 11]. Disease-causing microbial agents use different mechanisms to resist AMAs; the production of inactivating enzymes especially β-lactamases is the most important one. β-lactamases are a heterogeneous group of enzymes that can hydrolyze the β-lactam ring of the β-lactam AMAs. Once the drug molecule is hydrolyzed, it will not be active to kill or inhibit the growth of the bacteria. Extended-spectrum β-lactamases (ESBLs) are enzymes which are produced by bacteria and can hydrolyze penicillins, cephalosporins and monobactams. However, ESBLs are sensitive to β-lactamase inhibitors like clavulanic acid, sulbactam, and tazobactam and they cannot hydrolyze carbapenems and cephamycins. Bacteria that produce β-lactamases are usually MDR and are linked to life-threatening hospital or community-acquired infections [12, 13]. They are associated with a higher proportion of treatment failure and mortality than infections caused by non-ESBL producers [14–16]. LFE such as *E*. *coli* and *Klebsiella* spp. are the predominant ESBL producers [17], which are also included at the top of the list of priority pathogens for research and development of new antimicrobials by WHO [18].

The interaction among humans, animals, and the environment is very high in dairy farming. The farming community consumes the milk without appropriate processing and makes direct contact with the animal for milking or other husbandry activities [19]. The dairy farm sewage or slurry waste is an important source of infectious agents including AMR strains. The sewage can contaminate humans directly through contact, or indirectly through sewage-contaminated river water that is used for various purposes including growing vegetables that are consumed by humans or animals [20].

Dairy farming is an important source of income and food for many families in Ethiopia. On these farms, AMAs are widely used to increase milk production, promote growth, or prevent and control animal diseases such as mastitis and others. The indiscriminate use of AMAs can create selective pressure and accelerate the emergence and spread of AMR [21–23]. AMR dynamics, particularly MDR and ESBL in the human-dairy interface must be understood to develop evidence-based strategies for combating the associated problems. Determining the MDR-I is also a good tool for health risk assessment, which identifies if isolates are from a region of high or low antimicrobial use [24]. Therefore, this study was designed to determine the magnitude of MDR, MDR-I, and ESBL-producing LFE isolated from the human-dairy interface in northwest Ethiopia.

## Methodology

### Study area and design

**Fig 1:**
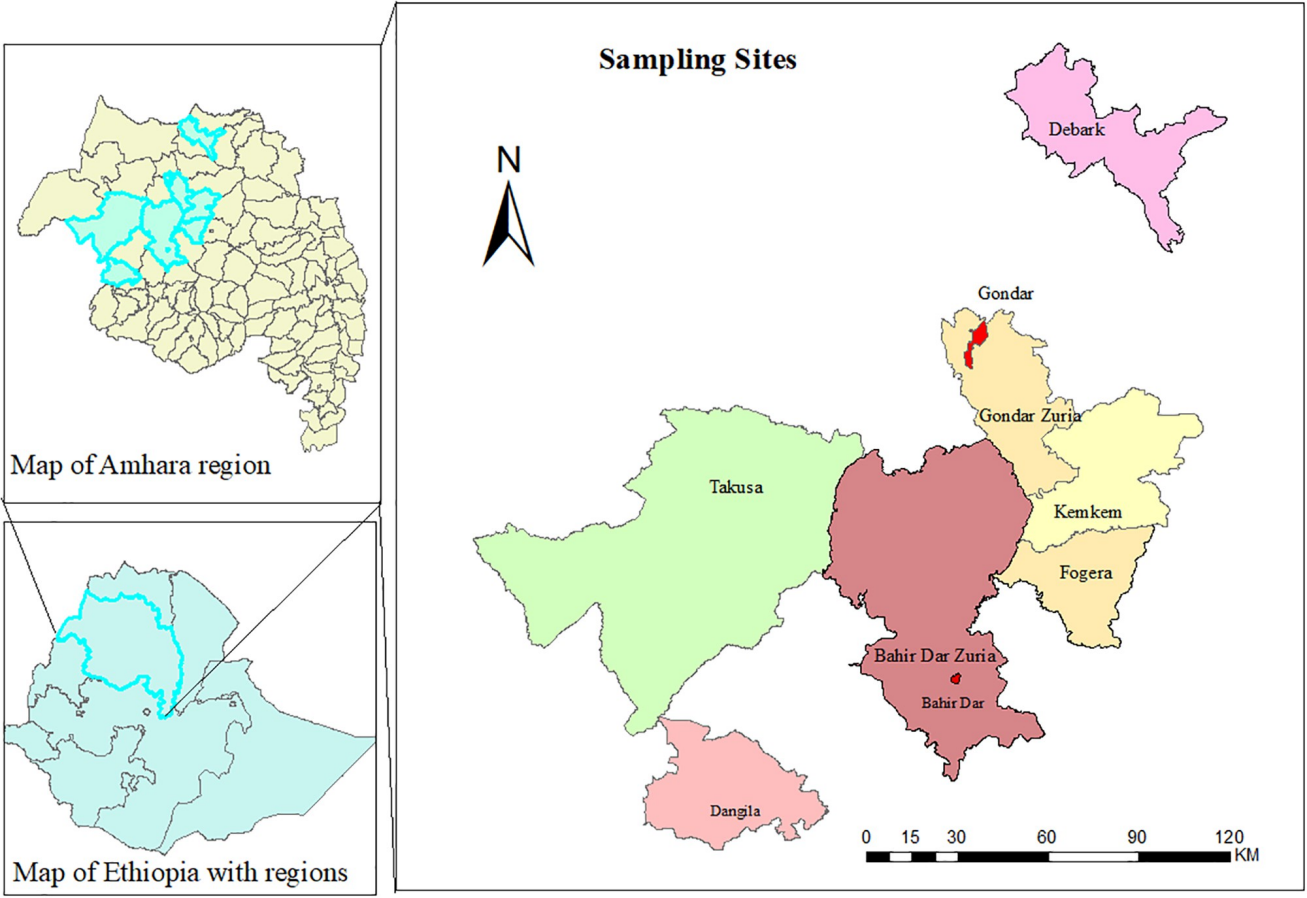
Map of the study areas (Sketched by ArcGIS maps (https://www.esrij.com/products/arcgis-field-maps/)).

The study was conducted in the Gondar-Bahir Dar milk shed in Amhara National Regional State, northwest Ethiopia (Fig 1). A cross-sectional study was conducted from June 2022 to August 2023. Samples were collected from dairy farms. First, a list of dairy farmers was obtained from livestock development offices in all selected sites. The dairy farms to collect samples were selected randomly by the lottery method. Pooled cows’ milk. which was ready to sell or consume, was collected for use in this study. Additionally, the milker’s hand swab, milk container swab, farm sewage, milker’s stool, and dairy cow feces were collected from each selected farm.

### Sample collection, transportation, processing, and bacteria identification

At the selected farms, approximately 50 ml milk samples were collected in sterile screw-cupped tubes. Swabs of the milk container and milker’s hand were collected using sterile cotton swabs, which were immersed in 9 ml of sterile buffered peptone water (Bio-Rad, Hercules, CA, USA). Samples (approximately 10 g, each) of dairy farm sewage, milker’s stool, and dairy cows’ fecal samples were collected aseptically using sterile sample collection tubes. All samples were transported in an icebox to the food safety laboratory of the University of Gondar, Gondar, Ethiopia, for bacteriological analysis. The samples were processed immediately after arrival at the laboratory. Sample collection was performed occasionally in the mornings during the study period.

*Escherichia coli* and other LFE were isolated as described in the FDA Bacteriological Analytical Manual [25] and ISO protocols [26] with slight modifications, as follows. The sample was homogenized and enriched in LFE selective broth (Oxoid Ltd, UK) at a 1:10 ratio (25 ml milk mixed with 225 ml broth, or 3 g of sewage, feces or stool mixed with 27 ml broth). Swabs in buffered peptone water were incubated directly at 37 °C for 24 hours, and then 1 ml of this fluid was added to 9 ml of broth). The sample-containing broth was incubated at 37°C for five hours and 42°C for about 36 hours and a loopful of the enriched sample was transferred to MacConkey agar (Oxoid Ltd) and incubated at 37°C for 24 hours. Three to five well-isolated and lactose fermenting colonies were subculture into Levine Eosin Methylene Blue agar (Neogen Culture Media, UK) and incubated at 37°C for 24 hours. After incubation, the bacteria were sub-cultured on tryptic soya agar (Sigma-Aldrich chemie GmbH, Germany) for biochemical tests including motility, indole, triple sugar iron, and citrate utilization. The colonies were further characterized by the analytical profile index (API) 20E (Biomerieux, France) test for identification [27].

### Antimicrobial susceptibility

The antimicrobial susceptibility test was performed using the Kirby-Bauer disk diffusion method [28]. Briefly, each bacterial isolate was grown overnight, some colonies were taken and added to a tube containing 0.85% sterile saline, vortexed, and the turbidity of the suspension was adjusted to match the 0.5%-McFarland standard. A sterile cotton swab was dipped into the suspension and then uniformly streaked over the entire surface of the Mueller-Hinton agar (MHA) (Sigma-Aldrich Chemie GmbH, Germany). Paper disks impregnated with a fixed concentration of antimicrobials (Oxoid Ltd, UK) were placed on the agar surface of agar and the plates (9 cm in diameter, six antibiotic disks per each) were incubated at 37 °C for 24 hours. After incubation, the zone of inhibition was measured in millimeters using a caliper and interpreted as “susceptible”, “intermediate” and “resistant” according to Clinical and Laboratory Standards Institute (CLSI) criteria [29]. Sixteen AMAs in nine antimicrobial classes were used to assess the antimicrobial susceptibility pattern (S1 Table). The bacterial isolates were grouped as MDR if they were resistant to at least one AMA in three or more classes [10, 30]. The MDR-I was calculated by dividing the number of antimicrobials to which the isolate was resistant by the total number of antimicrobials tested; a value of ≥ 0.2 indicates the high-risk area where antimicrobials are often used [31, 32].

### Detection of extended-spectrum beta-lactamase-producing bacteria

To detect ESBL production, the isolates were screened against ceftriaxone, cefotaxime, and ceftazidime using the Kirby-Bauer disk diffusion method and interpreted based on the CLSI guideline [29]. Bacterial isolates exhibiting a zone of inhibition equal to or less than 22 mm for ceftazidime, 25 mm for ceftriaxone, or 27 mm for cefotaxime were preliminarily identified as potential ESBL producers. These isolates were then marked for subsequent phenotypic confirmation. Phenotypic confirmation was conducted using a combined-disk diffusion test. An isolate that passed the screening test was emulsified in 0.85% sterile saline solution and its turbidity was matched with 0.5 McFarland standard and then inoculated on MHA by using sterile swabs. Cefotaxime (30 μg), and cefotaxime/clavulanic acid (30μg/10μg) disks were applied on the plate separately with at least 25 mm space between them and then plates were incubated at 37 °C for 24 hours. If there is ≥ 5 mm an increase in inhibition zone diameter for cefotaxime in combination with clavulanic acid as compared with the zone diameter of the tested cefotaxime alone, was considered as ESBL-producing isolates [29]. The genotypic confirmation was conducted by using multiplex PCR targeting the three ESBL-encoding genes namely *bla*_*TEM*_, *bla*_*SHV*_, and *bla*_*CTX-M*_ [33]. Table 1 depicts the primer pair sequence and amplicon length of the target genes.

**Table 1:** The name and sequence of primers used for genotyping confirmation.

Target gene	Primer Sequence	Amplicon length	References
*bla*_*TEM*_	F: 5’ TCGCCGCATACACTATTCTCAGAATGA	445	[34–37]
*bla*_*TEM*_	R: 5’ ACGCTCACCGGCTCCAGATTTAT		
*bla*_*SHV*_	F: 5’ ATGCGTTATATTCGCCTGTG	747	
*bla*_*SHV*_	R: 5’ TGCTTTGTTCGGGCCAA		
*bla*_*CTX-M*_	F: 5’ ATGTGCAGCACCAGTAAAGTGATGGC	593	
*bla*_*CTX-M*_	R: 5’ TGGGTAAAGTAAGTGACCAGAATCAGCGG		

F = forward, R = reverse

The DNA was extracted from each phenotypically confirmed ESBL-producing isolate by heat lysis. Briefly, 250 μl of the overnight incubated tryptic soya broth (Sigma-Aldrich chemie GmbH, Germany) with bacteria was centrifuged at 11,000 rpm for 3 minutes. The supernatant was discarded, and the pellets were washed with 1 mL sterile saline (0.85%). After centrifugation at 11,000 rpm for 3 minutes and discarding the supernatant, 100 μl of nuclease-free water was added to the cell pellets, which were vortexed to homogenize and boiled at 100°C for 10 minutes followed by chilling on dry ice for about 5 minutes. Finally, the debris was separated by centrifugation at 13,500 rpm for 5 minutes and the supernatant was taken and stored at -20°C until use as the DNA template. Amplification of target genes was carried out in a total volume of 20 μl reaction mixture consisting of 10 μl of Master mix (hot start GoTaq green Master mix, Promega USA), 0.75 μl of each of the primers (a total of 4.5 μl) (Table 1), 3.5 μl nuclease-free water and 2 μl template DNA. The genes were amplified in a thermocycler machine (MasterCycler, China) which was adjusted at initial denaturation of 95 °C for 15 minutes, with 30 cycles consisting of denaturation at 95 °C for 60 seconds, annealing at 60 °C for 40 seconds, and extension at 72 °C for 60 seconds and then followed by a final extension at 72 °C for 5 minutes. The amplified products were visualized after gel-electrophoresis using 1% agarose gel after staining with ethidium bromide. A 100-bp DNA ladder (Quick-Load ^®^ Purple DNA Ladder; Biolabs, England) was used as a molecular size marker to estimate the size of the PCR products. Amplicons were run at 140V for 50 minutes and visualized under an ultraviolet transilluminator then the image was taken using the gel documentation system (BioTop, Gel documentation system, China).

### Quality control

All bacteriological media were prepared based on the manufacturer’s instructions. Sterility tests were conducted after autoclaving of the media and all precautions were taken to avoid contamination during processing. *Escherichia coli* (ATCC 25922) and *Klebsiella pneumoniae* (ATCC 700603) strains were used as quality strain control during the running of each laboratory test.

### Ethical consideration

Ethical clearance was obtained from the University of Gondar Institutional Research Ethics Review Board (Ref. VP/RTT/05/60/2021). Data were collected in each farm after explaining the purposes of the study and oral consent was obtained from the owner or farm manager and the milker. The samples were coded without mentioning the name of the farm or the individual.

### Data management and analysis

The collected data were transferred to an Excel Microsoft spreadsheet and then to Statistical Package for Social Sciences (SPSS) software version 16 for analysis. Data were analyzed using a combination of descriptive and inferential statistical techniques (chi-square and analysis of variance). *P-*value < 0.05 was considered significant.

## Results

### Prevalence and multi-drug resistance patterns of lactose fermenting enterobacteriaceae

**Fig 2:**
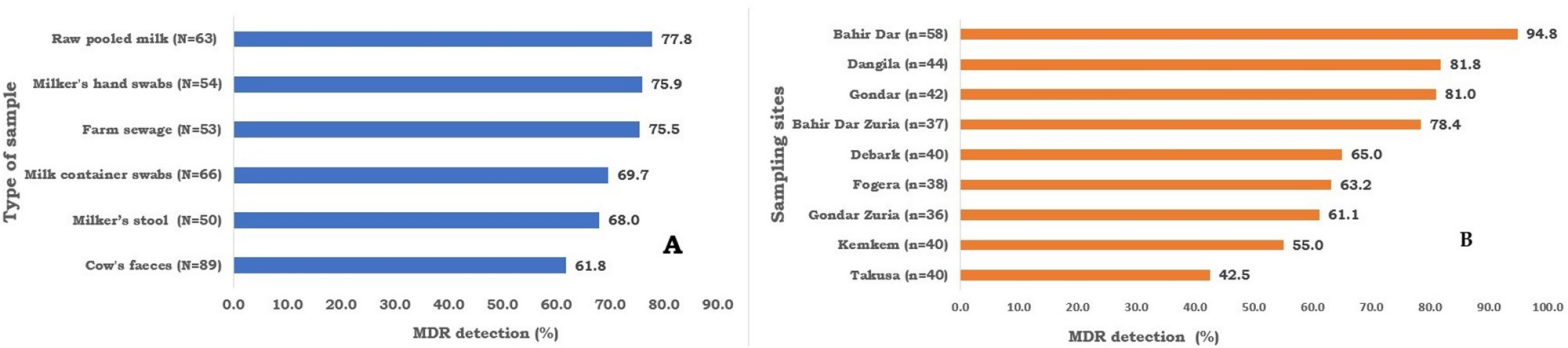
The multidrug resistance (MDR) detection of isolates per sample type (A) and sampling sites (B) (N = number of isolates tested).

In this study, a total of 375 bacteria were isolated from 362 samples consisting of raw pooled milk (58), milk container swabs (58), milker’s hand swabs (58), farm sewage (57), milker’s stool (47), and cow’s feces (84). Of these samples, 331 (91.4%) were positive for either of the LFEs. The prevalences of *E*. *coli*, *Citrobacter* spp, *Enterobacter* spp and *Klebsiella* spp were 71.3, 15.2, 10.2 and 6.9%, respectively. Among the bacterial isolates, 70.7% were MDR. Based on the type of bacteria, 73.0, 72.5, 65.5 and 60.0% of isolates of *Enterobacter* spp., *E*. *coli*, *Citrobacter* spp., and *Klebsiella* spp. were MDR, respectively. Fig 2 depicts the detection percent of MDR isolates per sample type and sampling sites. Despite the absence of statistically significant differences among sample types, a comparatively high proportion of MDR detection was observed in raw pooled milk (77.8%), milker’s hand swab (75.9%), and farm sewage (75.5%) samples. The difference in the detection of MDR based on the sampling sites was statistically significant *(P<0*.*05)* and the highest (94.8%) was observed among isolates from Bahir Dar and the lowest (42.5%) was detected in samples from Takusa.

The resistance pattern and number of classes of antimicrobials to which the isolates were resistant are shown in Table 2. A portion of the isolates (7.7%) exhibited no resistance to any of the tested antimicrobial classes, whereas other isolates demonstrated resistance to a range of one to nine different classes of antimicrobials.

**Table 2:** The resistance pattern of the isolates.

Number of classes of antimicrobials*	*E*. *coli* (N = 258), n (%)	*Citrobacter* spp (N = 55), n (%)	*Klebsiella* spp (N = 25), n (%)	*Enterobacter* spp (N = 37), n (%)	Over all (N = 375), *n (%)*
Resistant to none	20 (7.8)	4 (7.3)	2 (8.0)	3 (8.1)	29 (7.7)
Resistant to one	21 (8.1)	8 (14.5)	4 (16.0)	5 (13.5)	38 (10.1)
Resistant to two	30 (11.6)	7 (12.7)	4 (16.0)	2 (5.4)	43 (11.5)
Resistant to three	54 (20.9)	8 (14.5)	2 (8.0)	3 (8.1)	67 (17.9)
Resistant to four	52 (20.2)	9 (16.4)	2 (8.0)	5 (13.5)	68 (18.1)
Resistant to five	46 (17.8)	10 (18.2)	6 (24.0)	9 (24.3)	71 (18.9)
Resistant to six	24 (9.3)	4 (7.3)	3 (12.0)	4 (10.8)	35 (9.3)
Resistant to seven	10 (3.9)	4 (7.3)	2 (8.0)	4 (10.8)	20 (5.3)
Resistant to eight	1 (0.4)	0 (0.0)	0 (0.0)	2 (5.4)	3 (0.8)
Resistant to nine	0 (0.0)	1 (1.8)	0 (0.0)	0 (0.0)	1 (0.3)

(*the detailed resistance patterns for each antimicrobial are available in the supplementary document) (N = total isolate tested, n = number in that category, % = percent

### Multidrug resistant index

The overall average MDR-I of the bacterial isolates was 0.3, but 61.1% of the isolates showed MDR-I greater than or equal to 0.2 (Table 3). There was no statistically significant difference among bacteria genera or the type of samples. The average MDR-I was greater than 0.2 in all sampling sites except Takusa (0.18). The difference was statistically significant *(P<0*.*05)* and the average index was the highest (0.45) in isolates from Bahir Dar and followed by Gondar (0.38) and Bahir Dar Zuria (0.34) (Table 3).

**Table 3:** Multidrug resistance index and number isolates with greater than or equal to 0.2 index.

Variable	Categories	MDR Index			Number (%) of isolates ≥ 0.2	*P-value*
		Minimum	Maximum	Average ±SE		
Type of Bacteria	*E*. *coli (N = 258)*	0.00	0.75	0.31±0.01	161 (62.4)	0.71
	*Citrobacter* spp (N = 55)	0.00	0.81	0.30±0.22	31 (56.4)	
	*Klebsiella* spp (N = 25)	0.00	0.63	0.27±0.19	13 (52.0)	
	*Enterobacter* spp (N = 37)	0.00	0.69	0.33±0.03	24 (64.9)	
Types of Samples	Raw pooled milk (N = 63)	0.00	0.81	0.36±0.02	48 (76.2)	0.21
	Milk container swab (N = 66)	0.00	0.81	0.27±0.02	39 (59.1)	
	Milker’s hand swab ((N = 54)	0.00	0.69	0.30±0.02	30 (55.6)	
	Farm sewage (N = 49)	0.00	0.75	0.29±0.03	30 (56.6)	
	Milker’s stool (N = 50)	0.00	0.63	0.29±0.03	29 (58.00)	
	Cow’s feces (N = 89)	0.00	0.75	0.30±0.02	53 (59.6)	
Sampling sites	Dangila (N = 44)	0.00	0.69	0.29±0.03	28 (63.6)	0.00
	Bahir Dar (N = 58)	0.13	0.75	0.45±0.02	54 (93.1)	
	Bahir Dar Zuria (N = 37)	0.00	0.63	0.34±0.03	28 (75.7)	
	Fogera (N = 38)	0.00	0.63	0.26±0.03	19 (50.0)	
	Kemkem (N = 40)	0.00	0.69	0.22±0.03	18 (45.0)	
	Gondar (N = 42)	0.00	0.81	0.38±0.03	29 (69.0)	
	Gondar Zuria (N = 36)	0.00	0.63	0.24±0.03	17 (47.2)	
	Debark (N = 40)	0.00	0.81	0.31±0.03	25 (62.5)	
	Takusa (N = 40)	0.00	0.38	0.18±0.01	11 (27.5)	
**Overall (N = 375)**		**0.00**	**0.81**	**0.30±0.01**	**229 (61.1)**	

N = number of bacterial isolates, SE = Standard Error, % = percent

### Extended-spectrum beta-lactamase production among bacterial isolates

#### A. Phenotypic characterization

**Fig 3:**
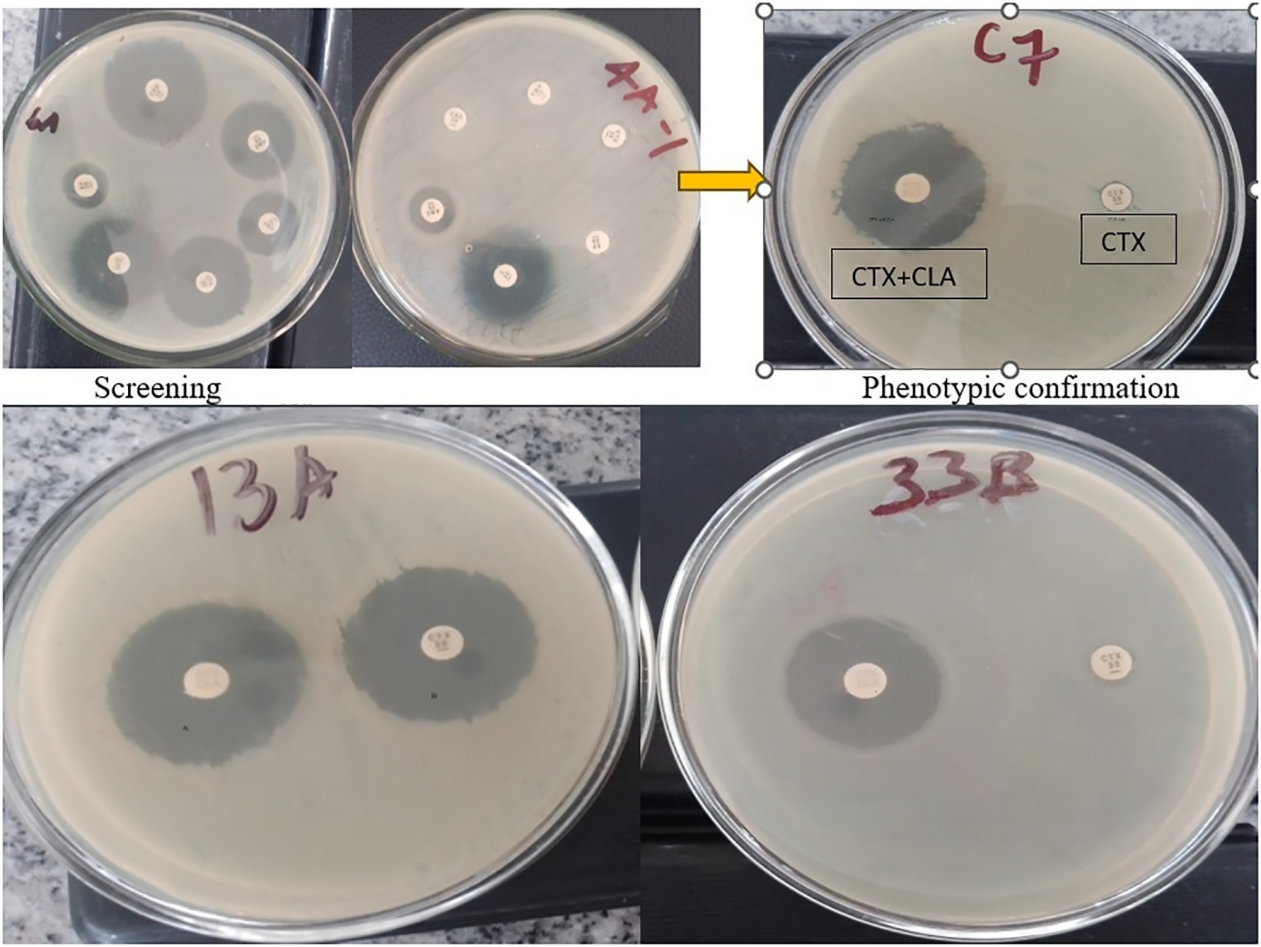
Screening and confirmation of beta-lactamase (ESBL) producing isolates (the upper two plates (6A and 4A-1) were at the screening stage in which 4A-1 fulfilled the screening criteria and right (C7) was the phenotypic confirmation, the disk on the left side was cefotaxime (CTX) and clavulanic acid (CLA) combination and the right was cefotaxime (CTX) alone where the difference in the zone of inhibition was greater than 5 whereas the picture on the bottom shows the ESBL negative isolate (13A) where the difference in the zone of inhibition was less than 5 and ESBLpositive isolate (33B) (the difference in the zone of inhibition was greater than 5 [10].

The overall detection proportion of ESBL production was 21.3% (N = 375) with a 95% confidence interval of 17.5 to 25.8%. Fig 3 depicts the screening and phenotypic confirmation of ESBL-producing bacterial isolates.

**Fig 4:**
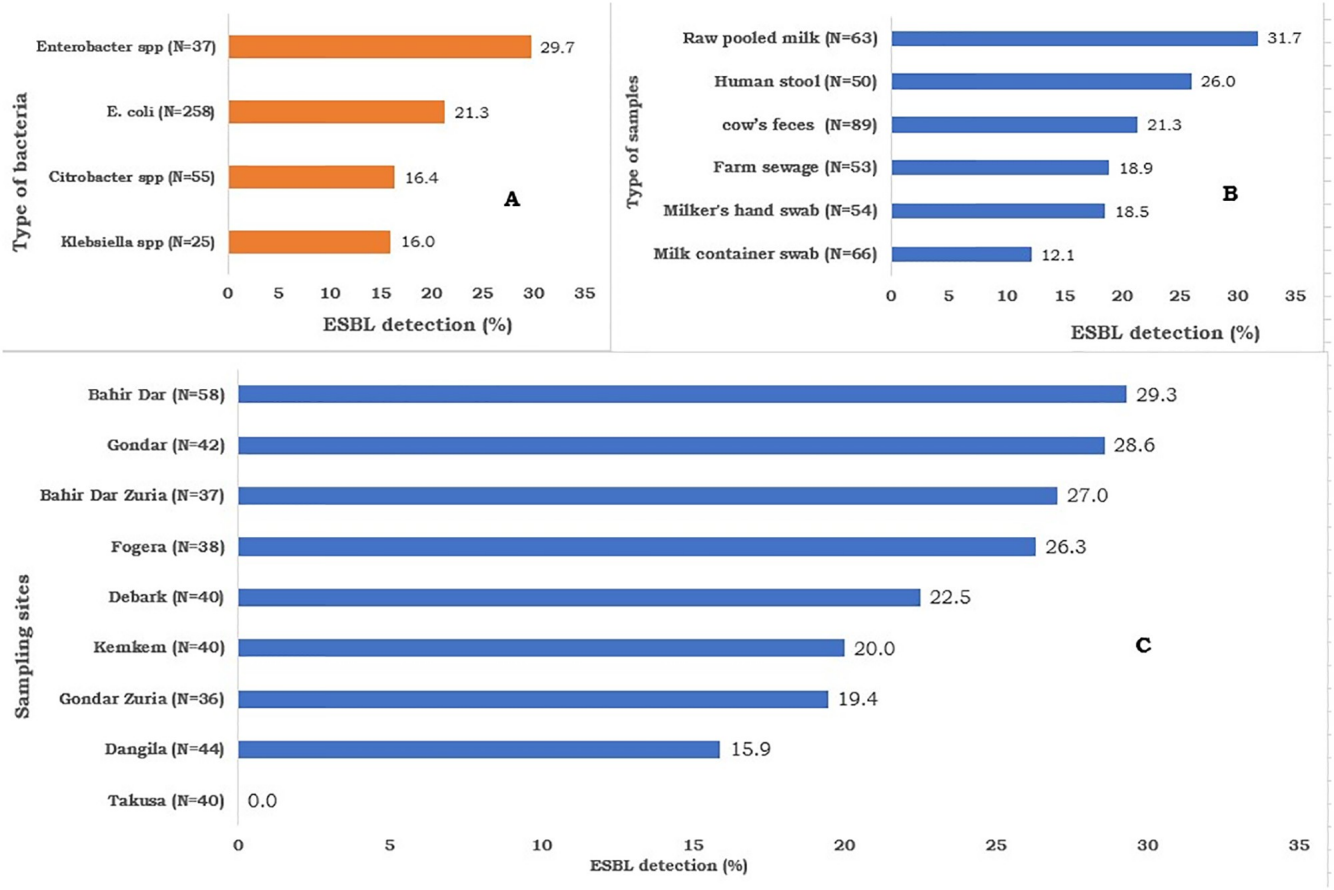
The detection proportion of extended-spectrum beta-lactamase-producing lactose fermenting Enterobacteriaceae (LFE) as per the type of bacterial isolates (A), type of samples (B), and sampling sites (C), N = number of isolates tested, % = percent.

Extended-spectrum β-lactamase (ESBL) production was detected in all isolates that belong to lactose fermenting Enterobacteriaceae. There was no statistically significant difference among bacterial isolates and type of samples. However, the detection was the highest among *Enterobacter* spp. (29.7%) and isolates from raw pooled milk (31.7%). The Difference among sampling sites was statistically significant (*P<0*.*05*); isolates from Bahir Dar (29.3%) and Gondar (28.6%) showed more ESBL detection than other sampling sites and none of the isolates from Takusa was positive for ESBL (Fig 4).

**Fig 5:**
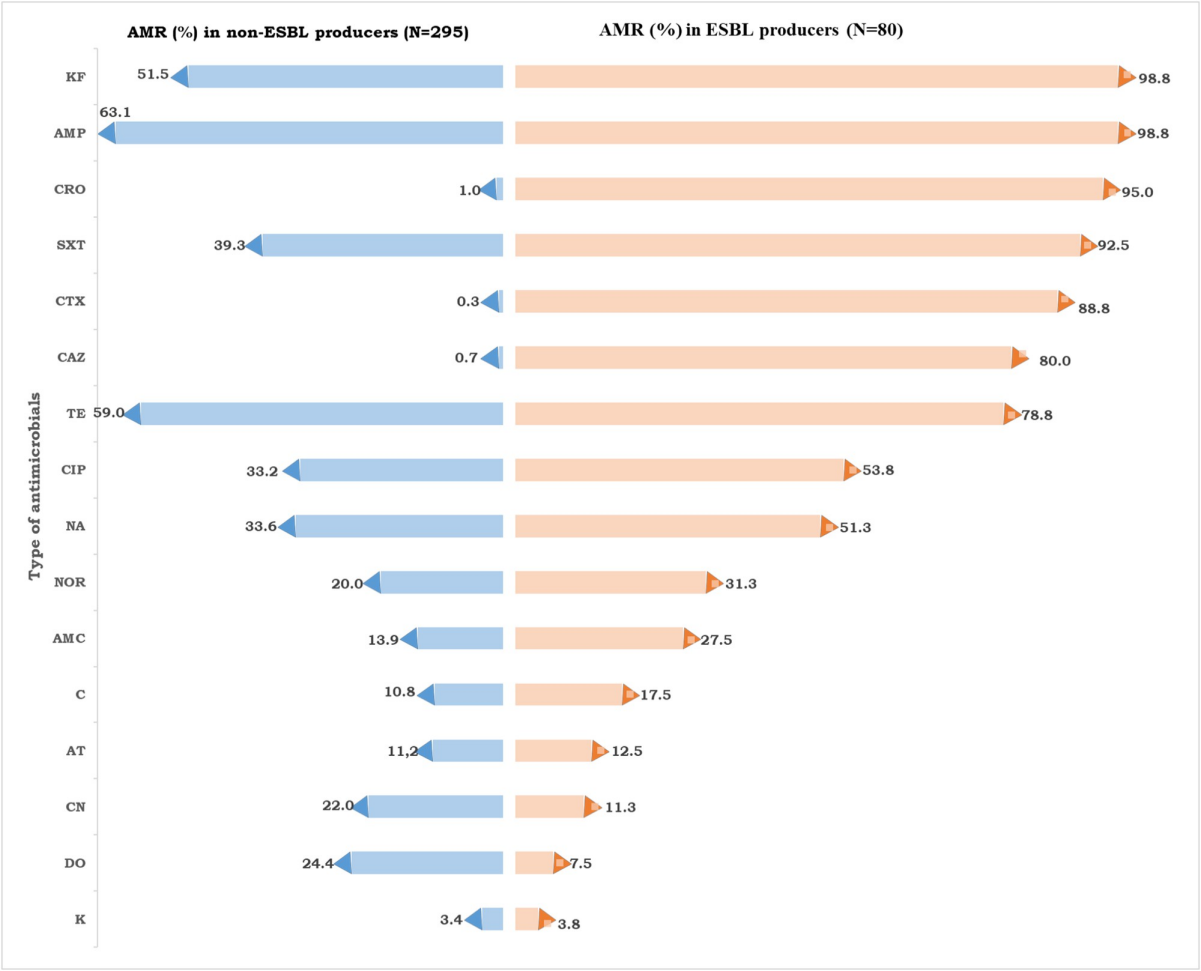
Comparison of the antimicrobial resistance (AMR) among extended-spectrum beta-lactamase (ESBL) non-producers and producers (AMP = Ampicillin, AMC = Amoxicillin-clavulanic acid, KF = Cephalothin, CRO = Ceftriaxone, CTX = cefotaxime, CAZ = ceftazidime, C = chloramphenicol, TE = Tetracycline, Do = Doxycycline, AT = Azithromycin, CN = Gentamicin, K = Kanamycin, NA = Nalidixic acid, NOR = Norfloxacin, CIP = Ciprofloxacin, SXT = Sulphamethoxazole-trimethoprim).

All ESBL-producing bacterial isolates were MDR. Fig 5 shows the comparison of the percent of resistant isolates among ESBL non-producing and producing LFE. It was observed that ESBL producers tend to be more resistant to the majority of antimicrobials than non-producers except doxycycline. The ESBL producers were highly resistant to cephalothin (98.8%), ampicillin (98.8), ceftriaxone (95.0%), sulphamethoxazole—trimethoprim (92.5%) and cefotaxime (88.8%) (Fig 5).

#### B. Genotypic characterization

**Fig 6:**
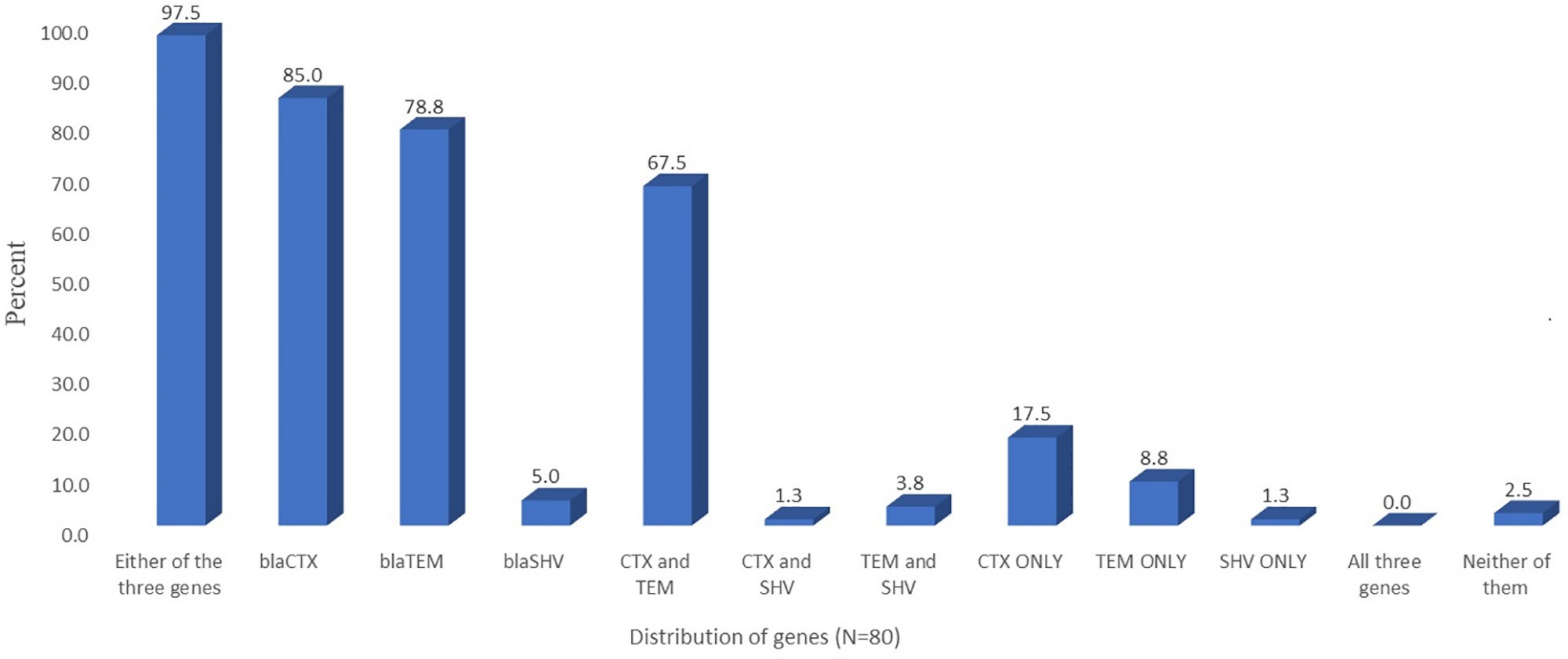
Distribution of extended-spectrum beta-lactamase (ESBL) encoding genes among phenotypically ESBL positive isolates.

**Fig 7:**
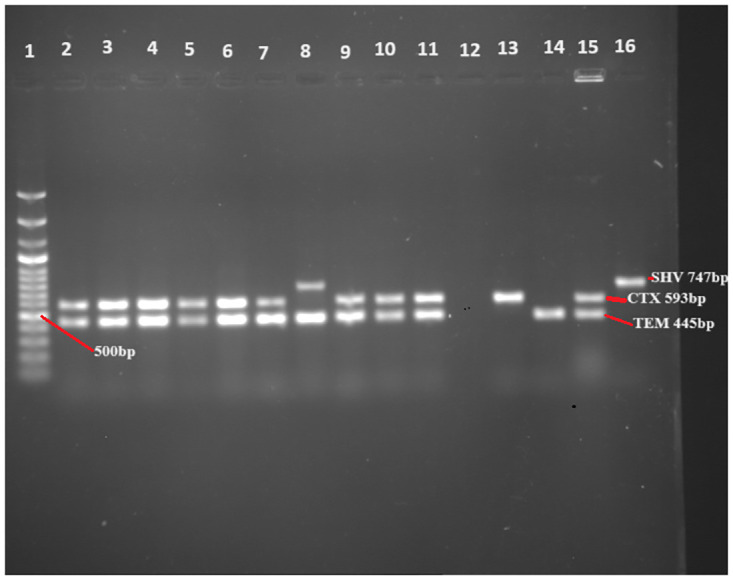
Representative gel picture (PCR products) in gel documentation (Lane 1 was ladder, lane 2,3,4,5, 6, 7, 9, 10, 11, 13, and 15 *bla*_*CTX-M*_ positive at 593 base pair (bp); Lane 2, 3, 4, 5, 6, 8, 9, 10, 11, 14, and 15 were *bla*_*TEM*_ positive at 445bp; Lane 8 and 16 were positive for *bla*_*SHV*_ gene at 747bp, 12 was negative for all whereas 16 was the positive controls,).

The majority of the isolates (97.5%) which were positive on the ESBL phenotypic confirmation test, were carrying at least one of the ESBL-encoding three target genes (*bla*_*TEM*_, *bla*_*SHV*_, and *bla*_*CTX*_). All three genes were detected among the bacterial isolates but *bla*_*CTX*_ (85.0%) and *bla*_*TEM*_ (78.8%) were the dominant (Fig 6). Fig 6 also depicts the distribution of the ESBL-encoding genes among phenotypically positive isolates whereas Fig 7 shows the representative PCR product used to detect the presence of target genes.

## Discussion

Antimicrobial resistance is becoming a big concern and threat to the public globally due to increasing the rate of resistance and the decline in the discovery of new antimicrobials to treat them [38]. There is a consensus that the problem has to be tackled by a multisectoral approach in a shared responsibility among human, animal and environmental sectors. To gear solutions in a one-health approach, evidence should also be generated in such a manner. In this study, a one-health approach was followed to generate evidence related to AMR from food (milk), animals, humans and the environment in the human-dairy interface where the interaction is usually high [19]. Three important parameters (MDR, MDR-I and ESBL production) were assessed to determine the status of AMR in LFE which were isolated from milk, milk container swabs, milker’s hand swabs, farm sewage, milker’s stool, and cow’s feces.

Multidrug-resistance strains are emerging as the result of continuous evolution and snowball effects mainly due to misuse or overuse of antimicrobials. The overall proportion of MDR bacterial pathogens in the current study was 70.7%. In line with this, the overall pooled prevalence of MDR bacterial infection in Ethiopia was 70.5% [39]. Another study reported a high prevalence of MDR bacterial infection (81.1%) in poultry farms in Gondar City, Northwest part of Ethiopia [30]. The high prevalence of MDR is related to the widespread misuse of antimicrobials [40]. Inappropriate use of antimicrobials is due to limited awareness, low attitude and malpractices which have been observed in the community, and among professionals in both human and animal health sectors [41–43].

*Escherichia coli* is a bacterium which is a common contaminant of food and water and acts as an indicator organism for the hygienic status of the food industry or water supply system. It is part of the normal flora of the gut. However, some strains are responsible for severe infections in humans and animals. The treatment of infection due to *E*. *coli* is becoming challenging if the organism contains MDR character. In this study, 72.5% of the *E*. *coli* isolates were MDR. Previously, the proportion of MDR *E*. *coli* isolates was 70.0% in Ethiopia [44]. Another study reported a 78.2% MDR prevalence of *E*. *coli* isolates from diarrheic patients in Ethiopia [45]. Up to 92.5% of MDR in *E*. *coli* was also reported by Bedasa *et al*. [46] in Bishoftu (central Ethiopia). The difference may be related to the variations in the intensity of antimicrobial use in different areas [47].

In this study, the proportion of MDR *Citrobacter* spp isolates was 65.5%. Previously, the proportion of MDR among *Citrobacter* spp was reported at 71% in Ethiopia by Tweldemedhin *et al*. [44]. A worldwide rise of MDR problems among *Citrobacter* spp was also reported by Jabeen *et al*. [48]. Among *Klebsiella* spp isolates, the proportion of MDR was 60.0% which was slightly lower than the previous report (68%) in Ethiopia [44]. About three-fourths (73.0%) of *Enterobacter* spp isolates showed MDR characteristics in this study. Multi-drug resistant *Enterobacter* spp infections are emerging in many parts of the world and a high prevalence (63.1%) of MDR among *Enterobacter* spp was also reported by Khademi *et al*. [49] in Iran.

The MDR index is a parameter that is used to assess whether the isolates originated from a site where antimicrobials are used in a large amount if it is greater than or equal to 0.2 [24]. The average MDR index of the isolates in this study was 0.3 which was an indication that antimicrobials were widely used in human-dairy interface in the area. The average MDR index was also greater than 0.2 in all sampling sites except Takusa. The index was the highest in Bahir Dar, Gondar and Bahir Dar Zuria sites. These may be related to the inappropriate use of antimicrobials and low awareness of professionals and the community on antimicrobial use and resistance in these areas as these were reported in previous studies [40, 43, 50]. These areas have relatively better access to antimicrobials due to the availability of suppliers and retailers.

The overall detection rate of ESBL in this study was 21.3%. Similarly, the proportion of ESBL enzyme-producing Enterobacteriaceae isolated from food handlers at the University of Gondar, northwest Ethiopia was 21.7% [51]. Another report indicated that the pooled prevalence of ESBL-producing Enterobacteriaceae in Ethiopia was 30% [33]. According to Zaatout *et al*. [52], the pooled prevalence of ESBL in Africa was 29.3% among Enterobacteriaceae isolated from wastewater being highest among isolates from hospital waste. They also reported that pooled prevalence in samples from farm and slaughterhouse sewage was 18.4%. These all are indicators that ESBL production among bacterial isolates is increasing and becoming a challenge for the treatment of bacterial infections.

In this study, all ESBL producer bacterial isolates were MDR which indicated that ESBL production is creating more chances for the organism to resist antimicrobials. A high rate of MDR (85.7%) among ESBL producers was also reported by Sivakumar *et al*. [53] in India. In addition, it had been observed that ESBL producers were proportionally more resistant to several antimicrobials than non-producers. Doxycycline was an exception which didn’t show such characteristics. Previously Sandhu *et al*. [54] and White *et al*., [55] reported that doxycycline exhibited efficacy for MDR isolates.

The ESBL production was detected in all common LFE isolates in this study. The proportion of ESBL enzyme-producing bacterial isolates were 29.7, 21.3, 18.2 and 16.0% among *Enterobacter* spp, *E*. *coli*, *Citrobacter* spp and *Klebsiella* spp, respectively. In contrast to this report, a higher pooled proportion of ESBL production among *Klebsiella* spp (61.8%), *E*. *coli* (41.2%) and other Gram-negative (41.9%) bacterial isolates either from human or animal was reported by Abayneh *et al*. [56] in Ethiopia and a 42.8% prevalence of ESBL-producing *E*. *coli* in dairy farms was reported by Braun *et al*. [57] in Egypt.

In this study, the proportion of ESBL-producing bacteria was considerably higher among bacteria isolated from raw pooled milk. This is a risk for consumers and emphasizes the need to increase hygienic milk production and proper treatment before consumption. Amare *et al*. [51] found that the isolation of ESBL enzyme-producing Enterobacteriaceae among food handlers was associated with the consumption of unpasteurized milk. A high proportion of ESBL enzyme-producing bacteria in milk samples as compared to fecal and environmental samples was also reported in Malaysia [58].

It is a common practice to use farm sewage for fertilizer or other activities without appropriate waste management which can be the source of AMR traits. In this study, the farm sewage harbors MDR (75.0%) and ESBL-producing LFE (17.3%). The pooled prevalence of ESBL enzyme production among Enterobacteriaceae in wastewater was 24.8% [52]. Another report in Nigeria indicated that the waste from the farm was one source of ESBL enzyme-producing bacterial contamination of vegetables [59]. Therefore, this evidence indicated that waste from farms can be the source of not only infection but also AMR bacteria.

The genes encoding for ESBL are usually localized in plasmids which makes them easily transferable among bacteria. The first β-lactamase was found in *E*. *coli* and termed TEM-1 after the name of the patient in which it was originally discovered (Temoneira) in Europe [60]. Before 1998, the dominant genes were *bla*_*TEM*_ and *bla*_*SHV*_ types. However, through mutation and gene exchanges, the diversity and the spectrum of β-lactamases expanded and several subfamilies and other families like *bla*_*CTX-M*_ have been discovered in recent years and become dominant in many parts of the world [61]. In this study, *bla*_*CTX-M*_ was found dominantly (85.0%) which was followed by *bla*_*TEM*_ type (Fig 6). In line with this, Kamaruzzaman *et al*. [58] reported that *bla*_*CTX-M*_ and *bla*_*TEM*_ genes were predominantly detected in samples from dairy cows, milk and farm environment in Malaysia. Other reports also showed the dominance of *bla*_*CTX-M*_ [52, 61–63].

The detection rate of the *bla*_*SHV*_ gene was the lowest (5.0%) in this study. An equivalent detection rate (8.1%) of the *bla*_*SHV*_ gene was also reported by Zhang *et al*. [64] in *E*. *coli* isolated from beef cattle in China. Other studies also reported low or no detection of the *bla*_*SHV*_ gene [53, 58].

## Conclusions and recommendations

The results of this study indicated that the three AMR parameters (MDR, MDR-I and ESBL production) were very high in northwest Ethiopia which signifies human-dairy interface is one of the important reserves and sources of AMR traits. The *bla*_*CTX-M*_ and *bla*_*TEM*_ were the most frequently detected genes in the ESBL-producing isolates. The output of this study is very important to design strategies for screening MDR and ESBL in different samples and provide more clues for developing standardized approaches for managing patients affected by ESBL-producing Enterobacteriaceae. To reduce the potential health hazard to the community, AMR mitigation strategies like prudent use of antimicrobials, awareness creation and infection prevention practices (hygienic milking practices, good husbandry practices, biosecurity and vaccination) have to be implemented in the area. The milk should be properly pasteurized or heat treated before consumption and farm sewage has to be managed before it is released into the environment or used for fertilizer or other practices.

## Strengths and limitations

We followed a new (one-health) approach by considering samples from humans, animals, food (milk), and the environment (sewage and swabs). They interact during the transmission of AMR which is helpful to design mitigation strategies. As the limitation and future activity, it would be better if the results were supported by advanced molecular techniques like gene sequencing.

## Supporting information

S1 File Raw data with codes, summary tables and figures (excel file with multiple sheets). (XLSX)

S2 File Resistant pattern of *Escherichia coli*. (XLSX)

S3 File Resistant pattern of *Citrobacter* spp. (XLSX)

S4 File Resistant pattern of *Klebsiella* spp. (XLSX)

S5 File Resistant pattern of Enterobacter spp. (XLSX)

S6 File API test, results and some typical colonies. (PDF)

S1 Data (ZIP)

S1 Raw images (PDF)

S1 Table Antimicrobials tested, their group, concentration, and cut points (CLSI, 2023). (DOCX)

## Abbreviations

AMAs: Antimicrobial Agents
AMR: Antimicrobial Resistance
CLSI: Clinical and Laboratory Standards Institute
ESBL: Extended-Spectrum Beta-Lactamase
FAO: Food and Agriculture Organization
LFE: lactose fermenting Enterobacteriaceae
LIC: Low-Income Countries
MDR: Multidrug Resistance
MDR-I: Multidrug Resistance Index
MHA: Mueller- Hinton agar
PCR: Polymerase Chain Reaction
WHO: World Health Organization
