# Supplementary material for: Multidrug-resistance and extended-spectrum beta-lactamase-producing lactose-fermenting enterobacteriaceae in the human-dairy interface in northwest Ethiopia
Source: PLoS One. 2024 May 21;19(5):e0303872. doi: 10.1371/journal.pone.0303872 (PMC11108214; doi:10.1371/journal.pone.0303872)
Supplement: S6 File — (PDF) [file pone.0303872.s006.pdf]

Major Steps of API E 20 test

1. A pure isolate of a bacterial suspension (in 85% saline) will be inoculated in each well and then incubated for 24 hours
2. Observe the color changes on the tubes some are automatic some colors come after adding reagents like Kovaks on indole

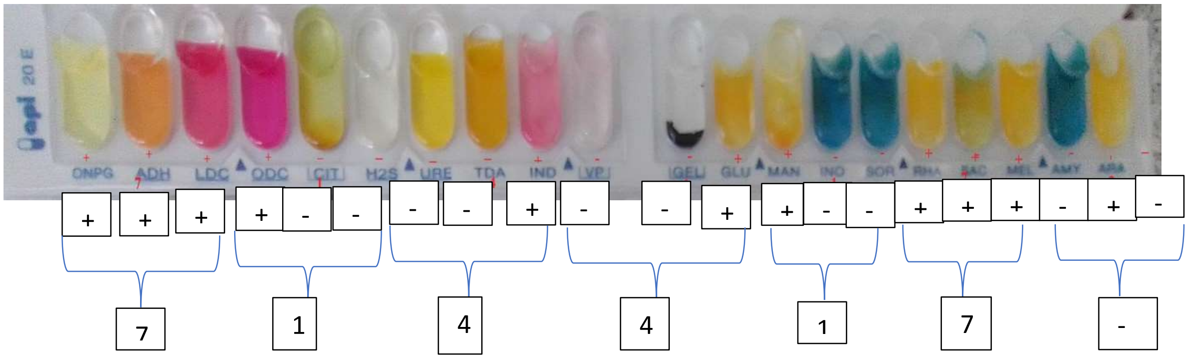

3. Convert to a seven-digit code like 7144172 (to produce digits, each tube is triplicate, the first tube will be given 1 if positive 0 if negative, the second 2 if positive 0 if negative, the last tubes will have a value of 4, the last triplicate will be complete by conducting oxidase test.
4. Insert the seven-digit code on the example above 7144172 on the API E-20 software. Like below,

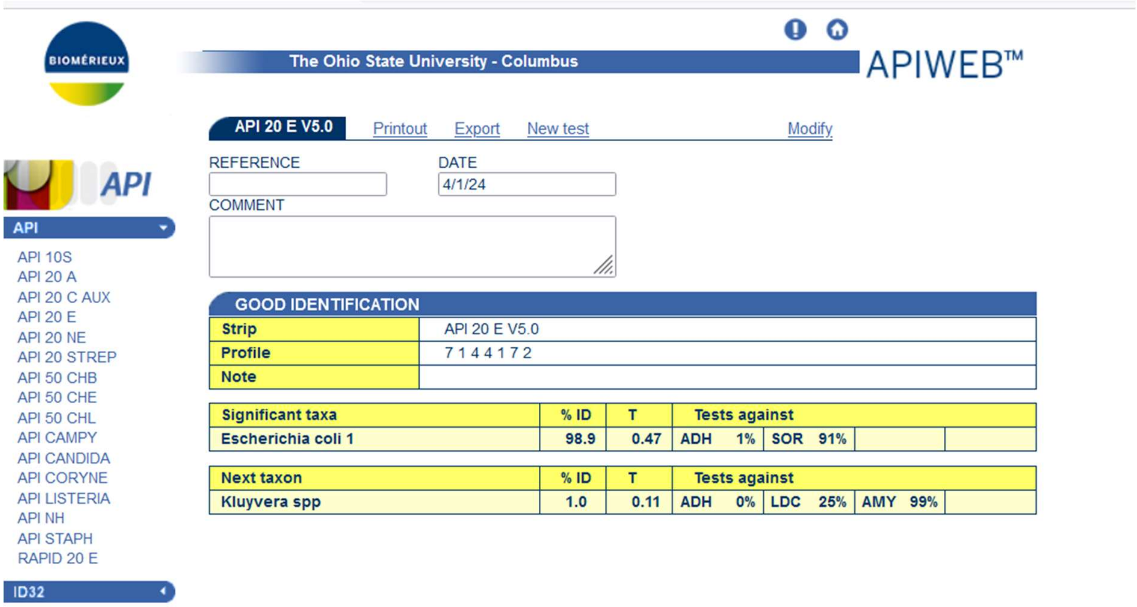

Hence, according to this, our test is 98.9% sure that it is *E. coli*,

Example 2

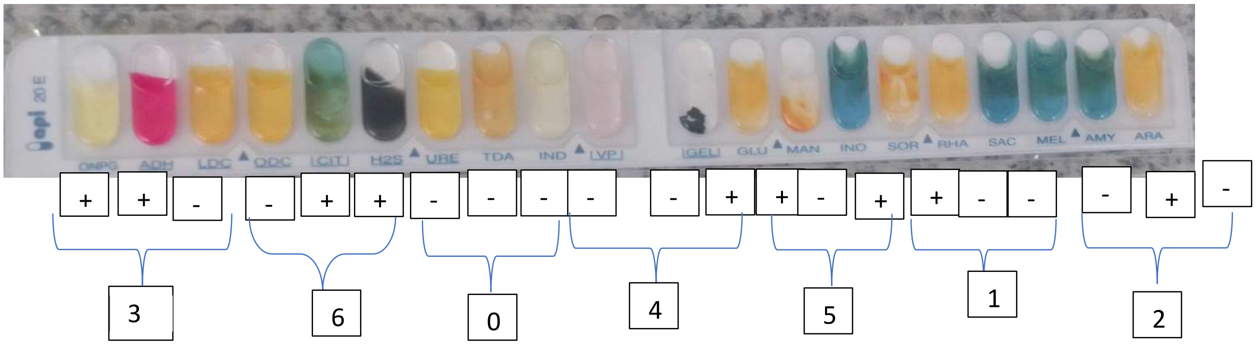

3604512

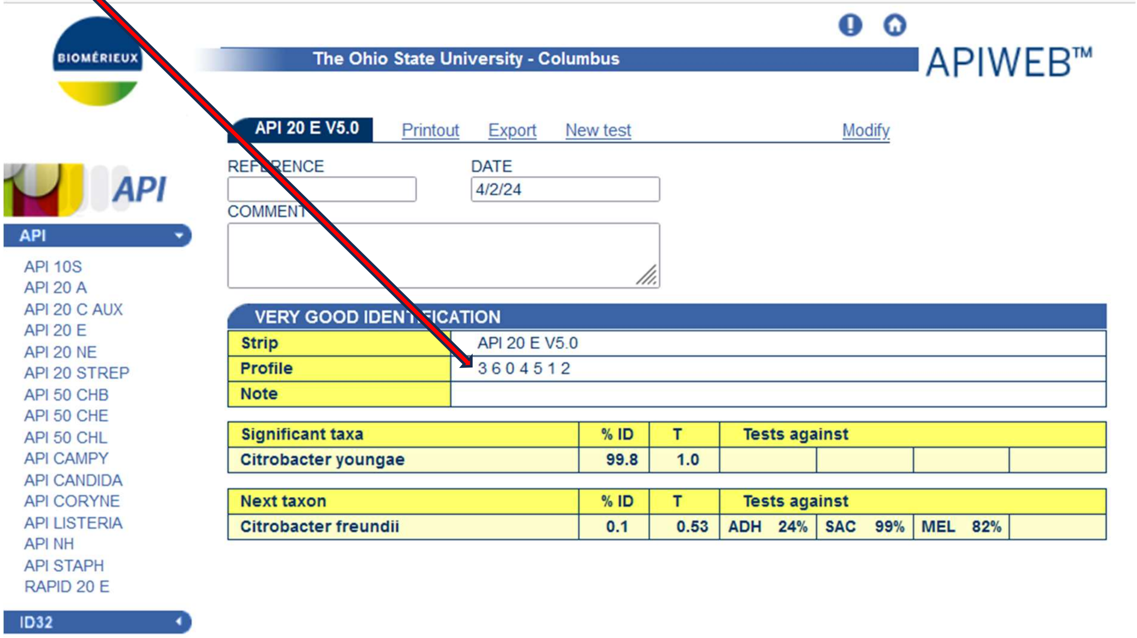
BIOMÉRIEUX

The Ohio State University - Columbus

APIWEB™

API 20 E V5.0 | Printout | Export | New test | Modify

REFERENCE

DATE 4/2/24

COMMENT

API

API 10S
API 20 A
API 20 C AUX
API 20 E
API 20 NE
API 20 STREP
API 50 CHB
API 50 CHE
API 50 CHL
API CAMPY
API CANDIDA
API CORYNE
API LISTERIA
API NH
API STAPH
RAPID 20 E

ID32

VERY GOOD IDENTIFICATION

Strip	API 20 E V5.0
Profile	3 6 0 4 5 1 2
Note	

Significant taxa	% ID	T	Tests against			
Citrobacter youngae	99.8	1.0				

Next taxon	% ID	T	Tests against			
Citrobacter freundii	0.1	0.53	ADH 24%	SAC 99%	MEL 82%	

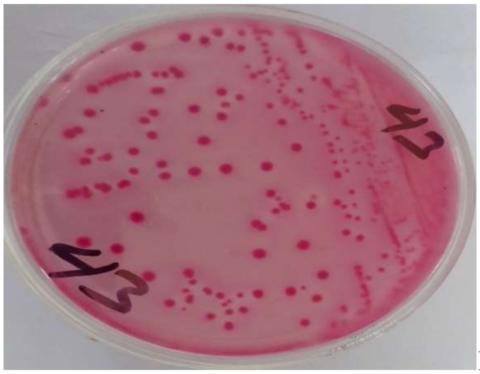

Lactose fermenting colonies of Enterobacteriaceae on MacConkey agar

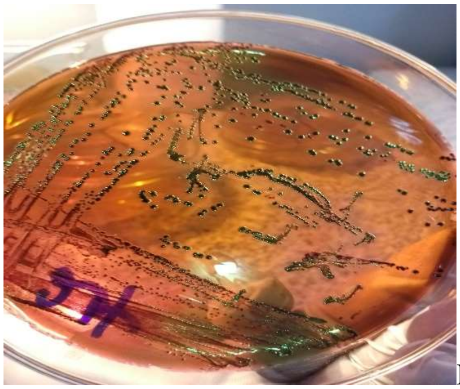
Metalic sheen production by some lactose fermenting on EMB agar
