# Supplementary material for: Multidrug-resistance and extended-spectrum beta-lactamase-producing lactose-fermenting enterobacteriaceae in the human-dairy interface in northwest Ethiopia
Source: PLoS One. 2024 May 21;19(5):e0303872. doi: 10.1371/journal.pone.0303872 (PMC11108214; doi:10.1371/journal.pone.0303872)
Supplement: S1 Data — (ZIP) [file pone.0303872.s007.zip › S1_raw_images.pdf]

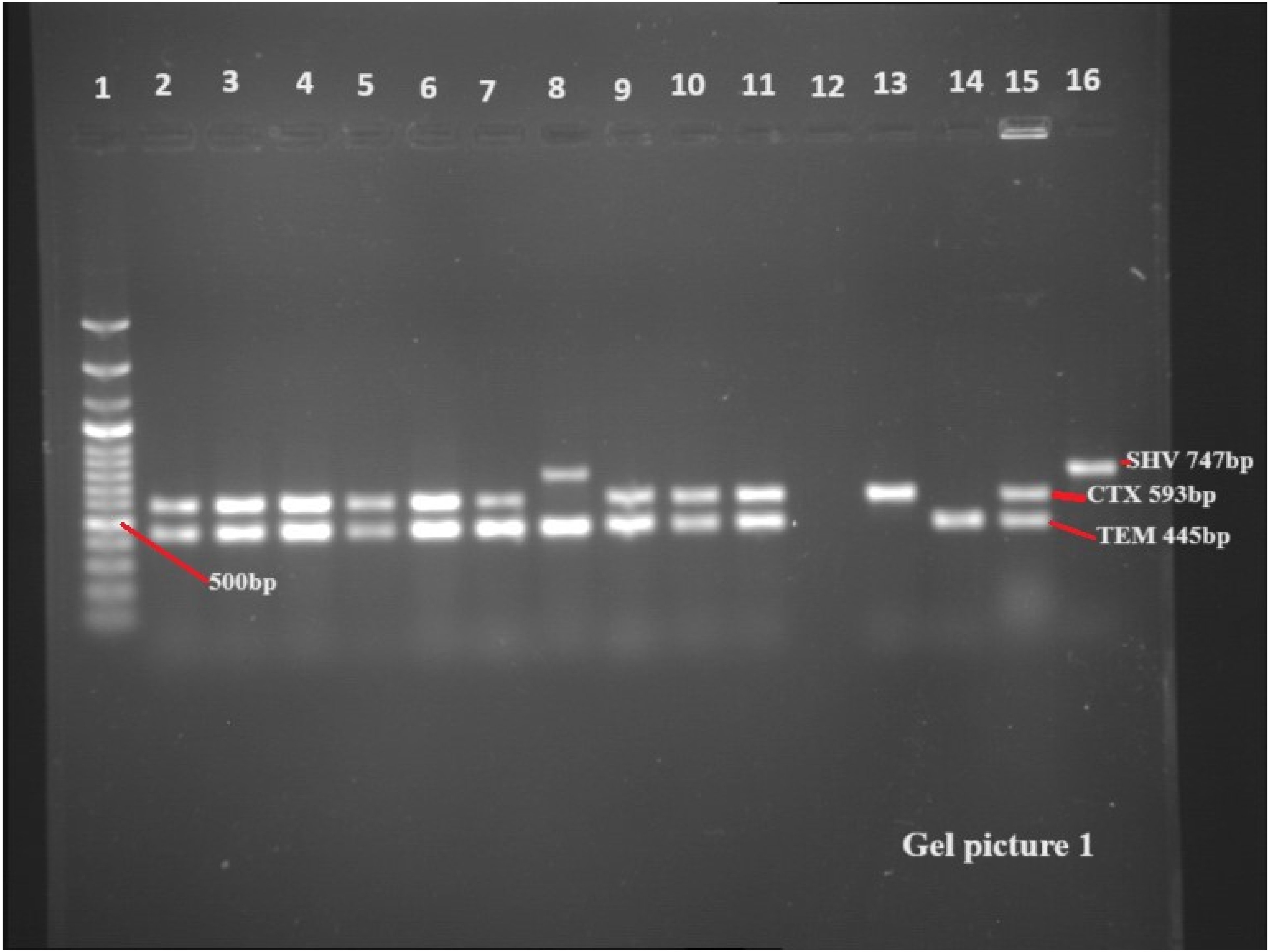

1 2 3 4 5 6 7 8 9 10 11 12 13 14 15 16
SHV 747bp
CTX 593bp
TEM 445bp
500bp
Gel picture 1

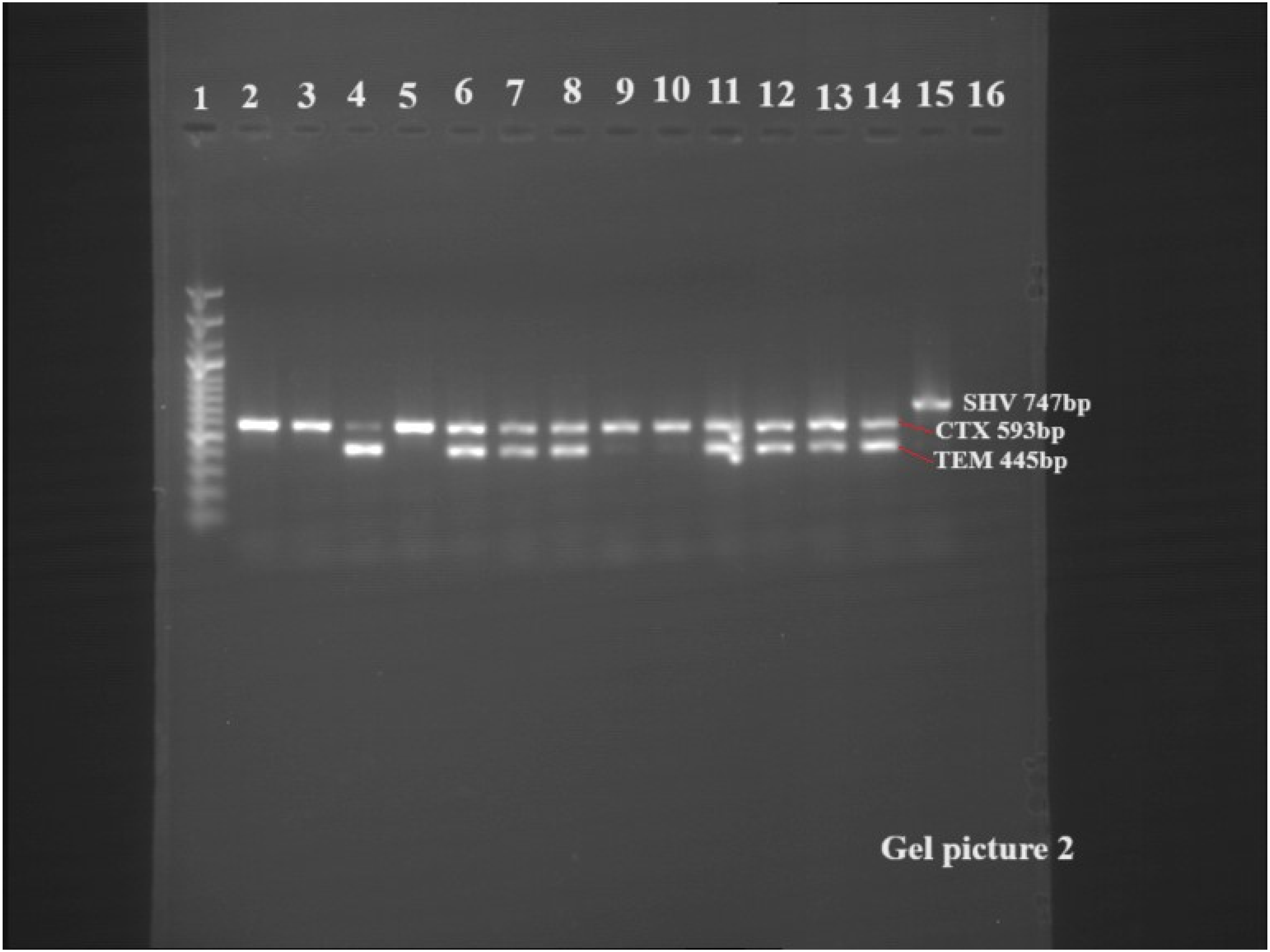
1 2 3 4 5 6 7 8 9 10 11 12 13 14 15 16
SHV 747bp
CTX 593bp
TEM 445bp
Gel picture 2

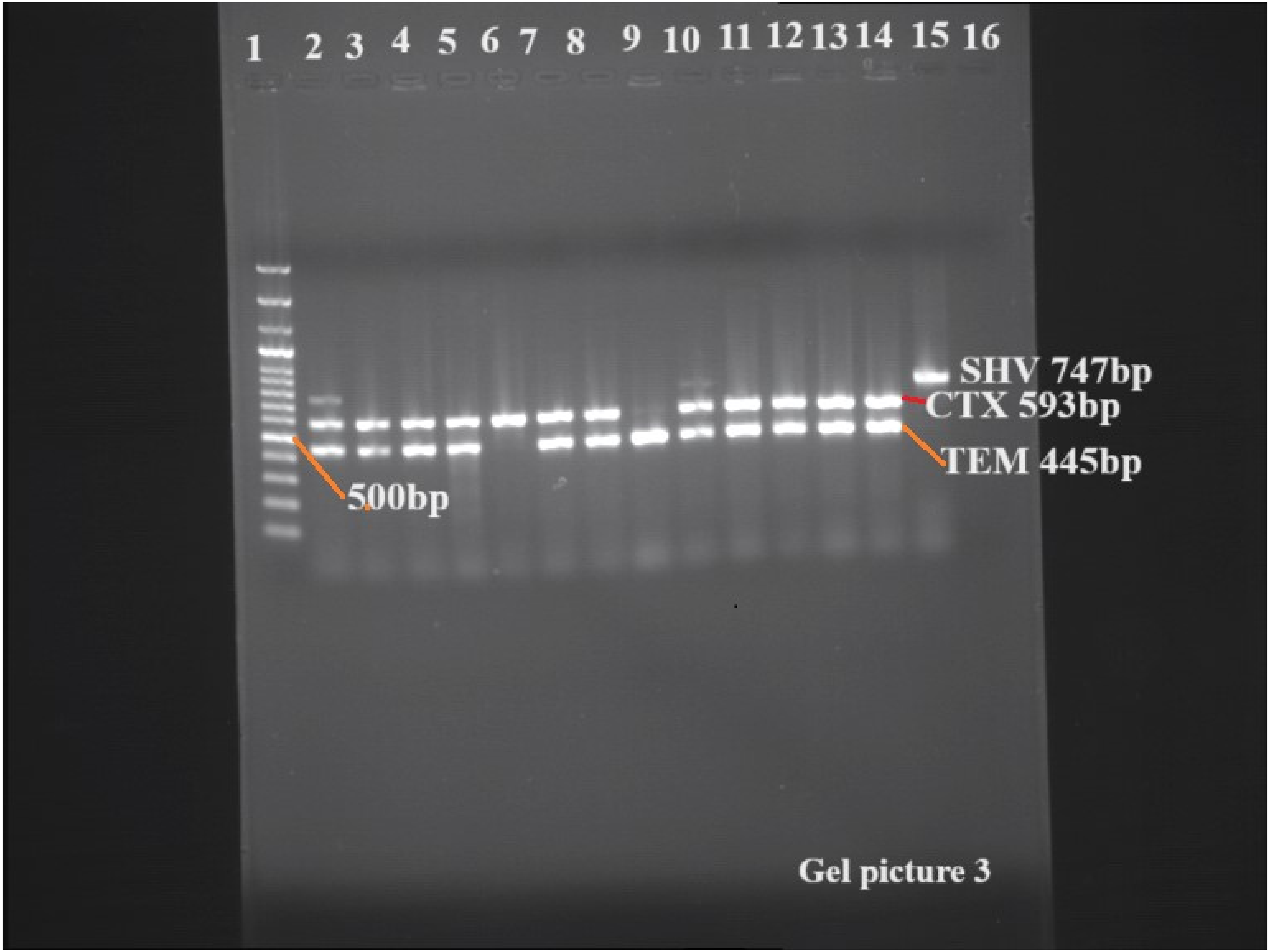
1 2 3 4 5 6 7 8 9 10 11 12 13 14 15 16
SHV 747bp
CTX 593bp
TEM 445bp
500bp
Gel picture 3

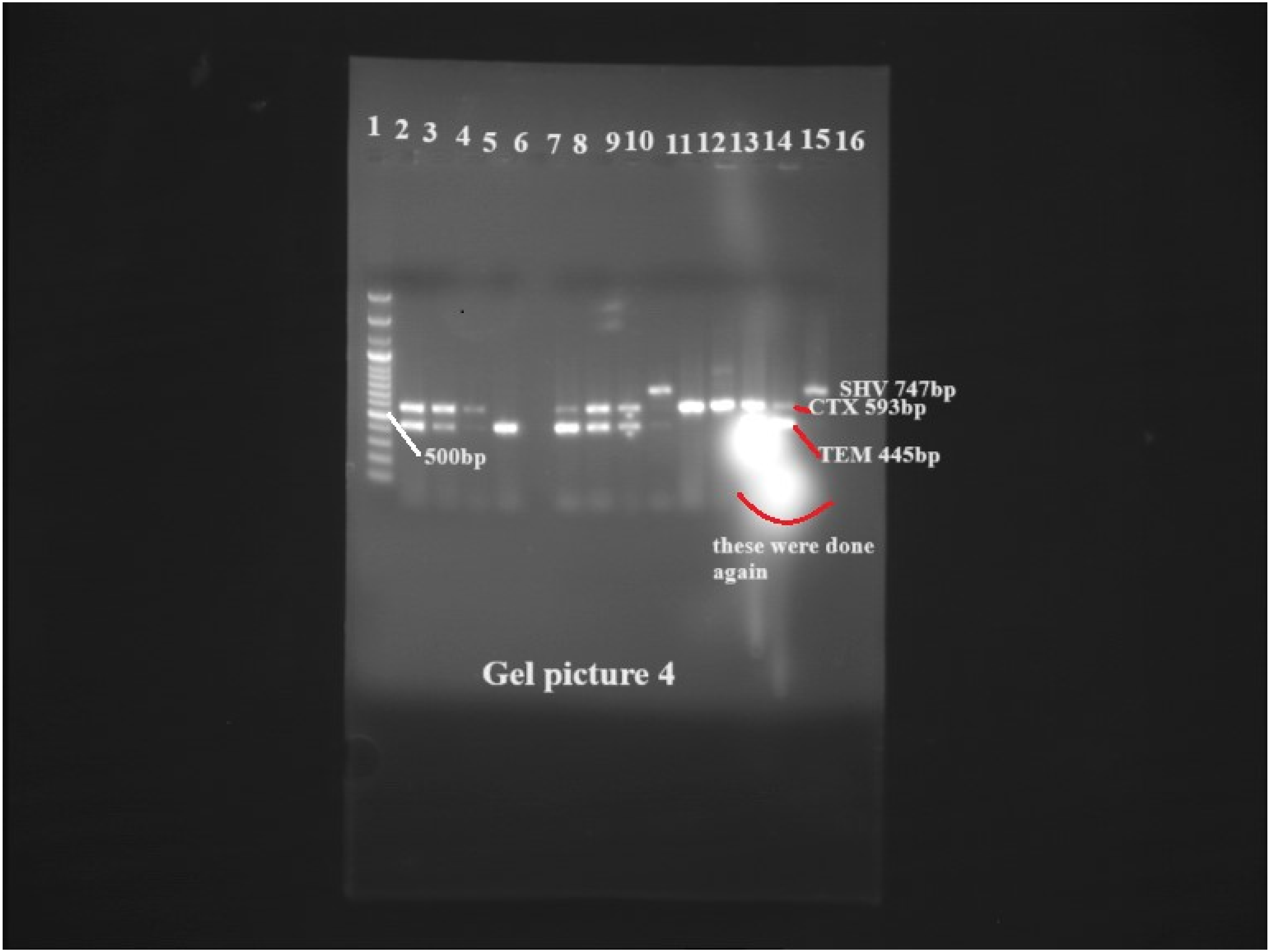
1 2 3 4 5 6 7 8 9 10 11 12 13 14 15 16
SHV 747bp
CTX 593bp
TEM 445bp
500bp
these were done again
Gel picture 4

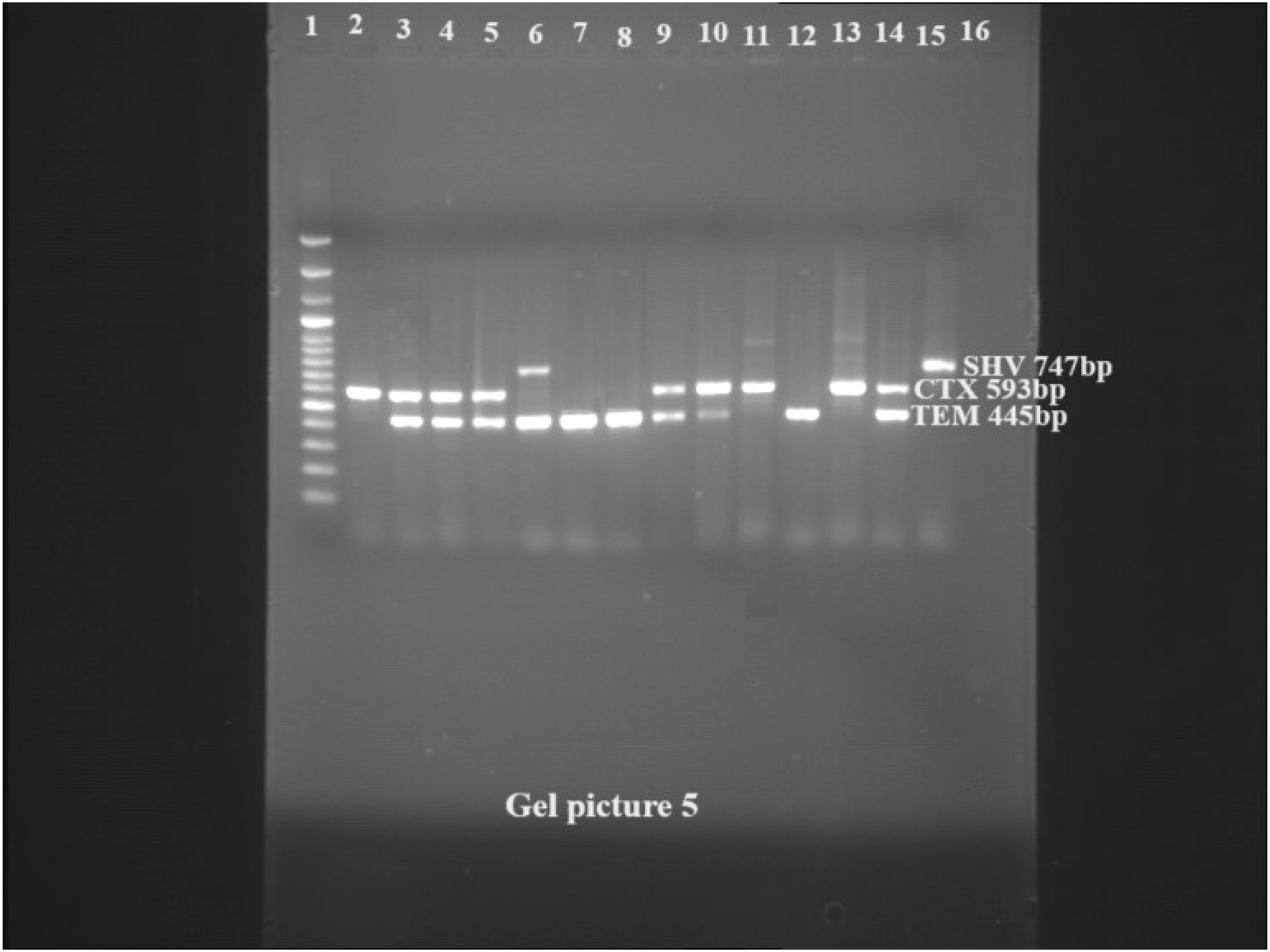
1 2 3 4 5 6 7 8 9 10 11 12 13 14 15 16
SHV 747bp
CTX 593bp
TEM 445bp
Gel picture 5

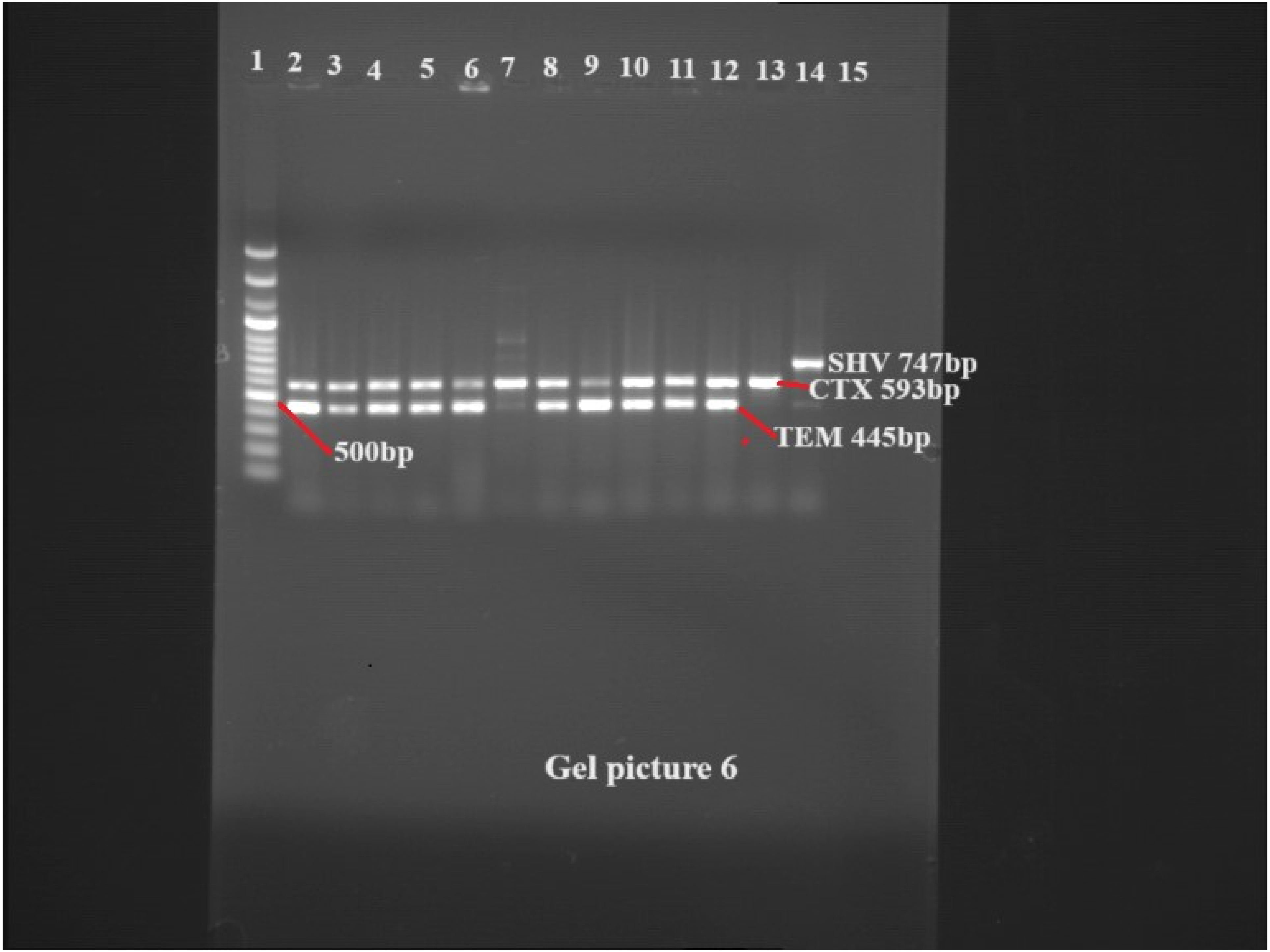
1 2 3 4 5 6 7 8 9 10 11 12 13 14 15
SHV 747bp
CTX 593bp
TEM 445bp
500bp
Gel picture 6

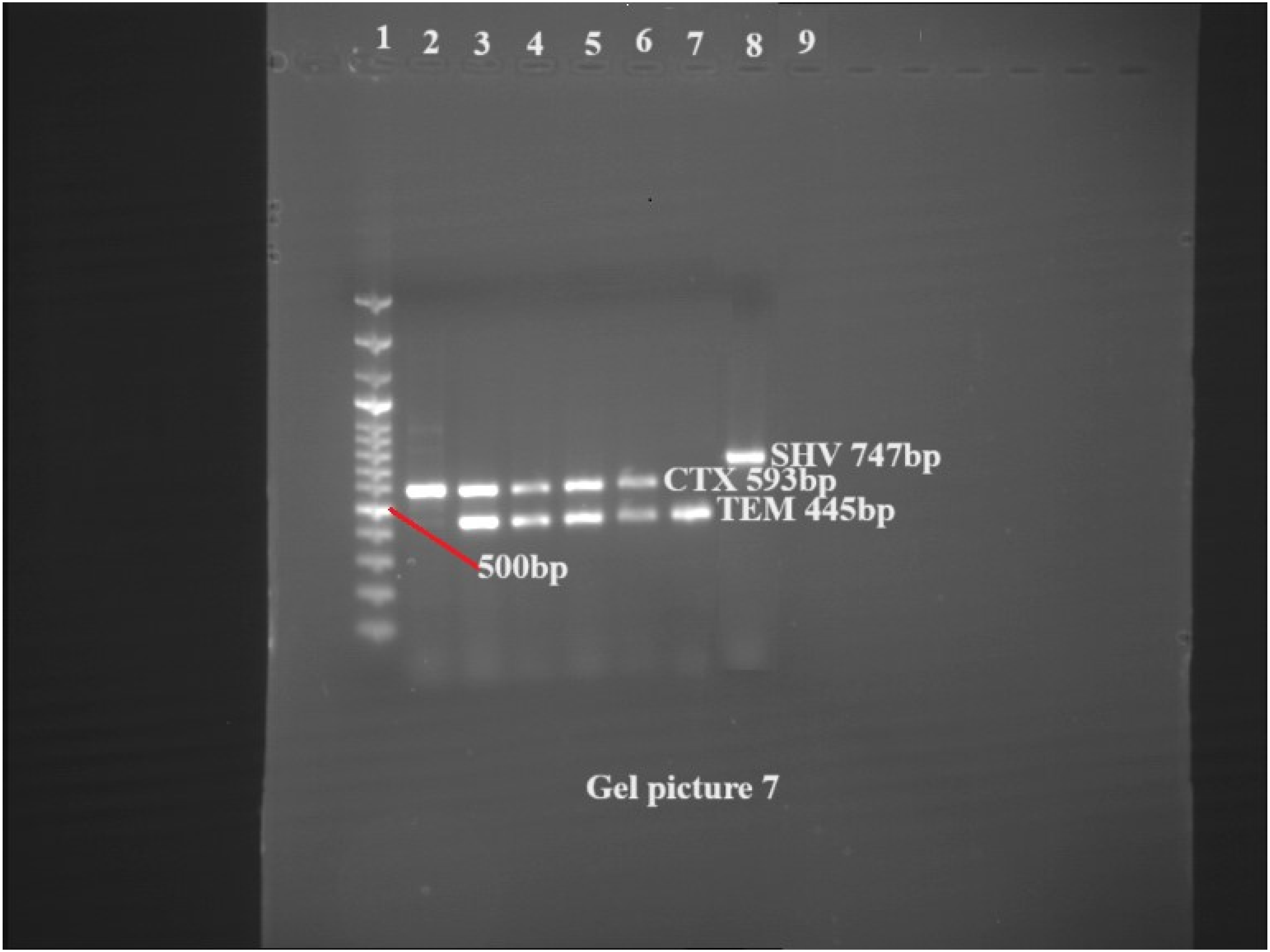

Gel picture 7

Interpretation of bands in the gel pictures

Pictures	order in the gel	code	blaSHV (747bp)	blaCTX (593bp)	blaTEM (445bp)	Remark
Gel picture 1	1	Ladder				
	2	DC2	0	1	1	1= positive
	3	DHS1	0	1	1	0=negative
	4	DF11	0	1	1	
	5	DC9	0	1	1	
	6	DH10a	0	1	1	
	7	DH10b	0	1	1	
	8	DM6	1	0	1	
	9	DM9	0	1	1	
	10	DS10	0	1	1	
	11	DF91	0	1	1	
	12	DF93	0	0	0	
	13	BM1	0	1	0	
	14	BM3	0	0	1	
	15	BC7a	0	1	1	
	16	Positive control	1	0	0	
Gel picture 2	1	Ladder				
	2	GF30	0	1	0	repeated
	3	BHS1	0	1	0	
	4	BHS6	0	1	1	
	5	BF12	0	1	0	
	6	BF13	0	1	1	
	7	BF23	0	1	1	
	8	BF31	0	1	1	
	9	BF33a	0	1	0	
	10	BF33b	0	1	0	
	11	BF42	0	1	1	
	12	DHS10	0	1	1	
	13	MBD4	0	1	1	
	14	MBD6	0	1	1	
	15	Positve control	1	0	0	
	16	Negative control	0	0	0	
Gel picture 3	1	Ladder				
	2	BD11a	1	1	1	
	3	BD15	0	1	1	
	4	BD19a	0	1	1	
	5	BD19b	0	1	1	
	6	BD23	0	1	0	
	7	BD25	0	1	1	
	8	BD34	0	1	1	
	9	DK12a	0	0	1	
	10	WM1a	0	1	1	

Pictures	order in the gel	code	blaSHV (747bp)	blaCTX (593bp)	blaTEM (445bp)	Remark
	11	WM1b	0	1	1	
	12	WM3	0	1	1	
	13	WM6	0	1	1	
	14	WHS16	0	1	1	
	15	Positve control	1	0	0	
	16	Negative control	0	0	0	
Gel picture 4	1	ladder				
	2	WHS17	0	1	1	
	3	WG23	0	1	1	
	4	Wsl28	0	1	0	
	5	Wsl29	0	0	1	
	6	WF37	0	0	0	
	7	AZm3b	0	1	1	
	8	AZm4a	0	1	1	
	9	AZm4b	0	1	1	
	10	AZc8a	1	0	0	
	11	AZh15	0	1	0	
	12	AZh17	0	1	0	repeated
	13	AZf33	0	1	1	repeated
	14	AZf34	0	1	1	repeated
	15	Positve control	1	0	0	
	16	Negative control	0	0	0	
Gel picture 5	1	ladder				
	2	DC7	0	1	0	
	3	DH8a	0	1	1	
	4	DH8b	0	1	1	
	5	DS7	0	1	1	
	6	BD36	1	0	1	
	7	DHS7	0	0	1	
	8	DHS9	0	0	1	
	9	DF72	0	1	1	
	10	GS18	0	1	1	
	11	GL25	0	1	0	
	12	GF29a	0	0	1	
	13	GF30	0	1	0	repeated
	14	MKM1	0	1	1	
	15	Positve control	1	0	0	
	16	Negative control	0	0	0	
Gel picture 6	1	Ladder				
	2	MKM3	0	1	1	
	3	MKM4	0	1	1	
	4	MKM5	0	1	1	

Pictures	order in the gel	code	blaSHV (747bp)	blaCTX (593bp)	blaTEM (445bp)	Remark
	5	MKM6a	0	1	1	
	6	MKM6b	0	1	1	
	7	MK25	0	1	0	
	8	DK4	0	1	1	
	9	DSH6a	0	1	1	
	10	DK21	0	1	1	
	11	DK22	0	1	1	
	12	DK28/1	0	1	1	
	13	DC6	0	1	0	
	14	Positive control	1	0	0	
Gel picture 7	1	Ladder				
	2	AZh17	0	1	0	repeated
	3	DHS6	0	1	1	
	4	AZf33	0	1	1	repeated
	5	AZf34	0	1	1	repeated
	6	BC7b	0	1	1	
	7	DS8	0	0	1	
	8	Positve control	1	0	0	
	9	Negative control	0	0	0	
