# Supplementary material for: Multidrug-resistance and extended-spectrum beta-lactamase-producing lactose-fermenting enterobacteriaceae in the human-dairy interface in northwest Ethiopia
Source: PLoS One. 2024 May 21;19(5):e0303872. doi: 10.1371/journal.pone.0303872 (PMC11108214; doi:10.1371/journal.pone.0303872)
Supplement: S1 Data — (ZIP) [file pone.0303872.s007.zip › S2 Table Antimicrobials tested.docx]

S1. Table. Antimicrobials tested, their group, concentration, and cutpoints (CLSI, 2023)

| **S/n** | **Class** | ***Antimicrobials*** | ***Code*** | **Concentration (**µg) | ***Resistance (mm)*** | ***Intermediate (mm)*** | ***Susceptible (mm)*** |
| --- | --- | --- | --- | --- | --- | --- | --- |
|  | Penicillins | Ampicillin | AMP | 10 | ≤13 | 14-16 | ≥17 |
|  | Penicillins and beta-lactamase inhibitor | Amoxicillin-clavulanic acid | AMC | 20/10 | ≤13 | 14-17 | ≥18 |
|  | Cephems | Cephalothin | KF | 30 | ≤14 | 15-21 | ≥22 |
|  |  | Ceftazidime | CAZ | 30 | ≤17 | 18-20 | ≥21 |
|  |  | Ceftriaxone | CRO | 30 | ≤19 | 20-22 | ≥23 |
|  |  | Cefotaxime | CTX | 30 | ≤22 | 23-25 | ≥26 |
|  | Phenols | Chloramphenicol | C | 30 | ≤12 | 13-17 | ≥18 |
|  | Tetracyclines | Tetracycline | TE | 30 | ≤11 | 12-14 | ≥15 |
|  |  | Doxycycline | DO | 30 | ≤10 | 11-13 | ≥14 |
|  | Macrolides | Azithromycin | AT | 15 | ≤12 | - | ≥13 |
|  | Aminoglycosides | Gentamicin | CN | 10 | ≤14 | 15-17 | ≥18 |
|  |  | Kanamycin | K | 30 | ≤13 | 14-17 | ≥18 |
|  | Fluoroquinolones | Nalidixic acid | NA | 30 | ≤13 | 14-18 | ≥19 |
|  |  | Norfloxacin | NOR | 10 | ≤12 | 13-16 | ≥17 |
|  |  | Ciprofloxacin | CIP | 5 | ≤21 | 22-25 | ≥26 |
|  | Folate pathway antagonists | Sulphamethoxazole-trimethoprim | STX | 21.75/1.25 | ≤10 | 11-15 | ≥16 |

mm=diameter of inhibition in millimeters
